# Long-Term Traffic-Related Exposures and Asthma Onset in Schoolchildren in Oslo, Norway

**DOI:** 10.1289/ehp.11491

**Published:** 2009-01-30

**Authors:** Bente Oftedal, Wenche Nystad, Bert Brunekreef, Per Nafstad

**Affiliations:** 1 Division of Epidemiology, Norwegian Institute of Public Health, Oslo, Norway;; 2 Institute for Risk Assessment Sciences, Utrecht University, the Netherlands;; 3 Julius Center for Health Sciences and Primary Care, University Medical Center Utrecht, the Netherlands;; 4 Institute of General Practice and Community Medicine, University of Oslo, Norway

**Keywords:** asthma, children, long-term exposure, nitrogen dioxide, NO_2_, respiratory symptoms, traffic

## Abstract

**Background:**

Whether there is a causal relation between long-term exposure to traffic and asthma development is so far not clear. This may be explained by inaccurate exposure assessment.

**Objective:**

We investigated the associations of long-term traffic-related exposures with asthma onset assessed retrospectively and respiratory symptoms in 9- to 10-year-old children.

**Methods:**

We collected information on respiratory outcomes and potential confounding variables by parental questionnaire in 2,871 children in Oslo. Nitrogen dioxide exposure was assessed by the EPISODE dispersion model and assigned at updated individual addresses during lifetime. Distance to major road was assigned at birth address and address by date of questionnaire. Cox proportional hazard regression and logistic regression were used.

**Results:**

We did not find positive associations between any long-term traffic-related exposure and onset of doctor-diagnosed asthma. An interquartile range (IQR) increase of NO_2_ exposure before asthma onset was associated with an adjusted risk ratio of 0.82 [95% confidence interval (CI), 0.67–1.02]. Handling early asthma cases (children < 4 years of age) with recovery during follow-up as noncases gave a less negative association. The associations for late asthma onset (≥ 4 years of age) were positive but not statistically significant. For current symptoms, an IQR increase of previous year’s NO_2_ exposure was associated with adjusted odds ratios of 1.01 (95% CI, 0.83–1.23) for wheeze, 1.10 (95% CI, 0.79–1.51) for severe wheeze, and 1.01 (95% CI, 0.84–1.21) for dry cough.

**Conclusions:**

We were not able to find positive associations of long-term traffic-related exposures with asthma onset or with current respiratory symptoms in 9- to 10-year-old children in Oslo.

Several studies have reported increased prevalence of asthma during the last decades, although recently stable or decreasing trends are seen in some countries (Eder et al. 2006). Simultaneously, car traffic has increased, and therefore several studies have investigated whether exposure to traffic-related air pollution is associated with respiratory outcomes (Heinrich and Wichmann 2004; Sarnat and Holguin 2007). These studies have demonstrated associations between short-term exposure and symptoms in children already diagnosed with asthma [U.S. Environmental Protection Agency (EPA) 2004]. However, it is less clear whether long-term exposure to air pollution causes asthma. Several studies have reported effects in favor of such a relationship (Brauer et al. 2007; Gauderman et al. 2005; Gordian et al. 2006; Guo et al. 1999; Hwang et al. 2005; Jerrett et al. 2008; Shima et al. 2002; Zmirou et al. 2004), others have reported effects in subpopulations only (Brauer et al. 2002; Clougherty et al. 2007; Hirsch et al. 1999; Kuehni et al. 2006; McConnell et al. 2002, 2006; Nicolai et al. 2003), and some have reported no effects (Diette et al. 2007; Krämer et al. 2000; Lewis et al. 2005; Nordling et al. 2008).

Exposure assessment of traffic-related air pollution has varied significantly among different studies, and misclassification of exposure, especially of self-reported exposure, may explain some of the inconsistent findings (Kuehni et al. 2006). To reduce misclassification, several researchers have noted the need to develop more accurate methods for exposure estimation, to reduce misclassification (Delfino 2006; Jerrett 2007; Jerrett et al. 2008; Sarnat and Holguin 2007). Recent pediatric studies have used a refined exposure metric, by combining spatial measurements of air pollution with geographic information system (GIS) data and regression modelling, and their results suggest stronger respiratory effects (Brauer et al. 2007; Gauderman et al. 2005).

In the present study we used a GIS including a dispersion model to calculate outdoor nitrogen dioxide concentrations at each child’s home addresses from birth in 1992–1993 to 2002; and all addresses throughout each child’s life have been used by Zmirou et al. (2004). Thus we were able to include several aspects of long-term exposure: exposure in early life, before asthma onset and previous year’s exposure in addition to distance from major road. Some studies have investigated the effects of early exposure on asthma in schoolchildren (Clougherty et al. 2007; McConnell et al. 2006; Zmirou et al. 2004) and in preschool children (Brauer et al. 2007; Nordling et al. 2008); and because asthma is expected to start early in life (Delfino 2006; Eder et al. 2006), this time aspect is important (Eder et al. 2006). Effects of lifetime exposure have also rarely been investigated (Clougherty et al. 2007; Zmirou et al. 2004). Similar to these studies, our exposure was present before asthma onset. Our main aim was to investigate the associations of long-term traffic-related exposures in early life and before onset with onset of doctor-diagnosed asthma assessed retrospectively in current 9- to 10-year-old children living in Oslo, Norway. In addition, we investigated the relations of previous year’s NO_2_ exposure and distance to major road with current respiratory symptoms.

## Methods

### Study population

A follow-up of the Oslo Birth Cohort—children born in Oslo in 1992–1993—was carried out in 2001–2002 (Nafstad et al. 2005). A cross-sectional study of all children born in 1992 and living in Oslo was carried out simultaneously. Of these children, 5,279 children with a valid address in Oslo in August 2001 were invited to participate, of whom 2,065 children were from the cohort. A total of 3,533 families (1,574 families from the cohort) accepted and completed the same self-administered questionnaire collected in the period between September 2001 and November 2002 (response rate was 66.9% overall and 76.2% from the cohort). The present study includes the children residing in Oslo in first year of life and last year before completing the questionnaire (*n* = 2,871), defined by nonmissing exposures. The study was approved by the Regional Ethics Committee and the Norwegian Data Inspectorate. Written informed consent was obtained from the parents.

### Traffic-related exposures

Outdoor air pollution was modeled by the Norwegian Institute for Air Research using EPISODE, a combined three-dimensional Eulerian/Lagrangian dispersion model based on prospectively collected data on emissions for several time periods, hourly meteorologic measurements, topography, and detailed background air pollution concentrations measured at regional background stations in the southern part of Norway (Oftedal et al. 2009). The model calculated hourly concentrations of NO_2_ for each square kilometer, whereas individual receptor points (varying from 3,813 in 1992 to 8,009 in 2002) got additional contributions from a line source model. This model calculated concentrations from busy roads up to 500 m from the road, depending on the number of vehicles (Oftedal et al. 2009). For exposure, addresses located within 30 m from a receptor point were assigned the concentration of the nearest point. Modeling of long-term averages has recently been evaluated by comparing modeled NO_2_ concentrations versus measurements from 10 monitoring stations in Oslo (Oftedal et al. 2009). We found low bias (fractional bias within ± 0.16 at monthly level) and scatter. Correlation seemed to increase with the length of the averaging period in winter (Pearson correlation *r* = 0.44 at monthly and 0.61 at seasonal level), and increased further when both seasons were pooled together (r = 0.76). These results indicate that the EPISODE model can represent long-term levels of local outdoor NO_2_ reasonably well.

With national identification numbers, the Norwegian Population Register provided a complete residential history for each child during the period 1992–2002. The municipality of Oslo gave the accompanying geographic coordinates, and we linked NO_2_ concentrations to each child’s home addresses throughout their lifetime. Several long-term exposures were included: early exposure in first year of life, average exposure from birth to asthma onset, and previous year’s exposure before completing the questionnaire. These exposures used 70% as cutoff for a nonmissing value.

In addition to modeled traffic-related exposure, we calculated distance to major road by GIS at birth address and the address at date of completing questionnaire. Major roads are main transport routes with any form of motor transport, especially heavy vehicles.

### Respiratory outcomes

Doctor-diagnosed asthma was defined as a “yes” to the question: “If the child has ever had asthma, has asthma been confirmed by doctor?” We combined that answer with one about timing—“If the child has had asthma, how old (in years) was the child when he/she got asthma?”—to designate onset of doctor-diagnosed asthma. Other respiratory outcomes were current wheeze, severe wheeze, and dry cough. “Current wheeze” entailed symptoms of wheeze in the preceding 12 months, “current severe wheeze” was four or more attacks of wheeze in the preceding 12 months, and “current dry cough” was dry cough during the night without simultaneous cold or other respiratory infections in the preceding 12 months.

### Covariates

Information on potential confounders was extracted from the questionnaire. We performed Cox regression analyses of asthma onset and linear regression analyses of modeled exposure for each covariate, shown in Table 1 except the present ones. All potential confounders were simultaneously included, and because none influenced the estimated associations, covariates that were statistically significant on 20% level were kept in the final model. These were sex, parental atopy, maternal smoking in pregnancy, paternal education, and maternal marital status at the child’s birth. Parental atopy was defined as a history of maternal or paternal asthma, hay fever, or eczema. Maternal marital status was obtained from the Medical Birth Registry in Norway. In addition, we added the indicator of the cohort population as effect modifier. We did not include skin prick test (SPT) positivity, defined as at least one positive SPT (Oftedal et al. 2007), because of an increase of 300 observations and no change in the estimated associations. SPT positivity was included only when estimating the interaction with SPT positivity. Performing logistic regression analyses of the current respiratory symptoms for single potential confounders, we applied the same criteria for inclusion into the final model as for asthma. In addition to keeping furry pets now, dampness problems now, and parental ethnicity, the same covariates as for asthma were included (Table 1). In addition to individual covariates, we also used census data in considering several contextual neighborhood socioeconomic factors at birth address in the analyses of asthma onset (Vassenden 1987): percentage of unmarried residents, households with income below the median, residents with primary education only, with manual class only, nondwelling owners, flat dwellers, and dwellers with less than one room per capita (Næss et al. 2007). In the analyses, these socioeconomic indicators were standardized by generating *z*-scores.

### Statistical methods

To investigate associations between long-term traffic-related exposures and asthma onset, we applied Cox proportional hazard regression model and adjusted for the covariates stated above. Each child’s observation time was censored at the year of moving to an address without information about NO_2_ exposure or at the date of completing the questionnaire. 1.9% (44 of 2,329) of the children were censored because of missing exposure measurements; four of these subjects reported doctor-diagnosed asthma from 3 to 7 years later. Asthma onset during first year of life was given as exposure for first year of life, because exposure before birth was not available. Exposure before asthma onset was included as a time-dependent covariate. Each time a risk set was created for a case with asthma onset, we recomputed the exposure for each individual in the risk set as the average exposure from the month after birth to the age (in years) of this onset. The proportional hazard assumption was checked by smoothing scaled Schoenfeld residuals over time. We also investigated early asthma onset from birth to 3 years of age and late onset from 4 years of age in separate analyses. In addition to the main analyses including all subjects reporting asthma onset as cases, we performed sensitivity analyses by including as noncases the subjects with early asthma onset who had recovered during follow-up. To study the functional form of the association, traffic-related exposures were also modelled by smoothing cubic splines using S-plus for Windows release 6.1 (Insightful Corporation, Seattle, WA, USA). We assessed effect modification by including interaction terms for exposure before asthma onset with sex, parental atopy, SPT positivity, and cohort indicator. In addition, we added standardized socioeconomic factors on neighborhood level, one indicator at a time. Results are expressed as risk ratio (RR) for asthma onset by an inter-quartile range (IQR) increase of traffic-related exposure. Incidence rate (IR) of asthma was calculated as number of cases divided by sum of person-years to asthma onset or to censoring. Logistic regression analyses were added for doctor-diagnosed asthma ever with results expressed as odds ratio (OR) for asthma by an IQR increase of traffic-related exposure.

To investigate associations of previous year’s exposure to NO_2_ and distance to major road with current symptoms, we applied multiple logistic regression analysis and adjusted for the covariates stated above. We assessed effect modification by including interaction terms for previous year’s exposure with parental atopy, SPT positivity, and cohort indicator. Results are expressed as OR for current symptoms by an IQR increase of traffic-related exposure. The analyses were performed using SPSS for Windows release 14.0.1 (SPSS Inc., Chicago, IL, USA).

## Results

### Characteristics of the study population

Table 1 shows that most characteristics were distributed equally among all children with questionnaire information only and among all children with both traffic-related exposure and questionnaire information. The exceptions are cohort indicator, parental ethnicity, and dampness problems in early life, with fewer children from the cohort, slightly fewer western (defined as western Europe, United States, Canada, Australia, or New Zealand as birth country) parents, and slightly more children with dampness problems in the questionnaire population. Comparing children who had both traffic-related exposures and questionnaire information (*n* = 2,871) with the analysis population without missing covariates (*n* = 2,329) showed that these populations were similar except for more western parents (85.6%), slightly more atopic parents (63.4%), and more children from the cohort (61.7%) in the analysis population. Comparing the children with both traffic-related exposures and questionnaire information from the cohort population with the noncohort population showed that the cohort subjects had more western, more atopic, and more educated parents, and had slightly more cohabitant mothers and slightly fewer single mothers at birth. Currently, they also kept more furry pets, had slightly less wall-to-wall carpeting, and fewer smoking parents.

### Distribution of traffic-related exposures

The traffic-related exposure levels are presented in Table 2. The annual residential outdoor NO_2_ levels were highest and had the widest range in first year of life. The mean NO_2_ level was 39.3 μg/m^3^ in first year of life and declined to 25.2 μg/m^3^ in previous year before questionnaire. In the first years of life, the mean NO_2_ levels were similar among children with onset of doctor-diagnosed asthma and the rest of the population (38.4 vs. 39.4 μg/m^3^ in first year of life and 30.2 vs. 31.2 μg/m^3^ in second year of life). The distributions were similar in the populations with and without missing covariates, in both sexes, in children both with and without SPT positivity, and for all paternal education levels (data not shown). The levels in the noncohort population were higher than in the cohort (mean levels 42.5 and 36.5 μg/m^3^ in first year of life, respectively), and the median levels were 42.3 μg/m^3^ in the nonatopic parents and 38.5 μg/m^3^ in the atopic parents. Other traffic-related pollutants in Oslo also modeled by EPISODE were particulate matter with aerodynamic diameter < 10 μm and < 2.5 μm (PM_10_ and PM_2.5_). These pollutants are correlated with NO_2_ (*r* = 0.79–0.91).

The distance to major road was lower at birth than at address by 10-year follow-up. The distributions were skewed to the right and median distance to major road was 403.6 m at birth address and 458.5 m at 10-year follow-up.

### Incidence and prevalence of respiratory outcomes

**Figure 1:**
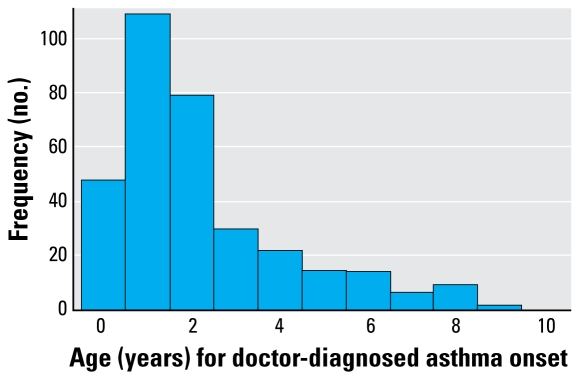
Frequency distribution of age (years) for doctor-diagnosed asthma onset (*n* = 327 cases).

Of the children’s parents, 11.8% reported asthma onset; IR = 0.014 cases per person-year (Table 3). Asthma was more incident in boys than girls (IR = 0.018 and 0.010 cases per person-year, respectively). Among children with onset of doctor-diagnosed asthma compared to the rest of the population, more children had atopic parents (73.9% versus 57.4%), more had a positive SPT (38.6% vs. 22.1%), more were male (63.7% vs. 49.5%), and more had a mother who smoked during pregnancy (22.2% vs. 15.3%). Similarly, of those with asthma onset, slightly fewer children had a parent with > 12 years education compared to the rest (51.9% vs. 59.4% for maternal and 53.1% vs. 61.3% for paternal education). Moreover, 92.3% of those who reported asthma onset also reported episodes of wheeze ever. The median age of asthma onset was 1 year, and 71.3% reported onset before 3 years of age (Figure 1), whereas 29.6% of these children had recovered at 4 years of age. Among the children with early asthma onset compared to those with later onset, fewer children had a positive SPT (35.1% vs. 53.3%), more had western parents (84.6% vs. 66.7%), and more had a parent with > 12 years education (53.6% vs. 44.8% for maternal and 54.8% vs. 45.5% for paternal education).

Of the current respiratory symptoms, 14.5% of the children’s parents reported wheeze, 4.8% reported severe wheeze, and 19.3% reported dry cough (Table 4). All current symptoms were more prevalent in boys than girls (17.1% vs. 11.7% in boys vs. girls for current wheeze).

### Associations between traffic-related exposures and respiratory outcomes

The estimated associations between long-term traffic-related exposure and doctor-diagnosed asthma are presented in Table 3, both crude and adjusted models. We did not find positive associations between any traffic-related exposure and asthma onset, but a tendency of negative associations with NO_2_ exposure and no associations with distance to major road. We found similar results when adding children who did not live in Oslo in previous year of life to the study population (*n* = 3,243) [adjusted RR = 0.80; 95% confidence interval (CI), 0.67–0.96 for an IQR increase of NO_2_ exposure before asthma onset and 0.98 (95% CI, 0.91–1.06) for an IQR increase of distance to major road]. The exception was late asthma onset, which produced positive but not statistically significant associations. The sensitivity analysis included those with early asthma onset who recovered during follow-up as noncases, and gave less negative associations [adjusted RR 0.92 (95% CI, 0.70–1.22) for an IQR increase of NO_2_ exposure before asthma onset, 0.85 (95% CI, 0.60–1.20) for early onset, and 1.08 (95% CI, 0.66–1.76) for late asthma onset]. The proportionality assumption was satisfied in all Cox proportional hazard regression models.

The interactions with exposure before asthma onset (sex, parental atopy, SPT positivity) were not statistically significant (*p* = 0.101 for sex), except for cohort indicator (*p* = 0.011). Stratified by sex, an IQR increase of NO_2_ exposure before asthma onset was associated with adjusted RR of 0.73 (95% CI, 0.56–0.95) (95% CI) in boys and 1.05 (95% CI, 0.74–1.49) in girls. Similarly, stratified by cohort indicator, the association was 0.66 (95% CI, 0.50–0.86) in cohort population and 1.11 (95% CI, 0.79–1.56) in noncohort population. Adjusting for contextual socioeconomic factors produced only minor changes in the estimated association (data not shown).

The estimated associations between traffic-related exposures and current respiratory symptoms are presented in Table 4, both crude and adjusted models. We found positive associations, although none were statistically significant; neither was any interaction with previous year’s exposure to NO_2_ statistically significant.

## Discussion

Long-term traffic-related exposures were not positively associated with onset of doctor-diagnosed asthma or with current respiratory symptoms in 9- to 10-year-old children in Oslo, Norway.

We aimed to assess associations between development of childhood asthma and traffic-related air pollution exposure in children’s home environment before disease development, by timing exposure before the development of disease. So far, few studies have been able to do so (Clougherty et al. 2007; McConnell et al. 2006; Nordling et al. 2008; Zmirou et al. 2004). We were not able to find any associations supporting the idea that the long-term traffic-related levels at historical residences in Oslo in 1992–2002 increased the risk of developing asthma among the participants. Several studies have shown positive associations between air pollution and asthma-related symptoms, supporting the presence of acute respiratory effects of air pollution both in healthy and asthmatic individuals (U.S. EPA 2004). For long-term exposure, some studies have reported positive associations with symptoms in schoolchildren (Gauderman et al. 2005; McConnell et al. 2006; Nicolai et al. 2003; Pierse et al. 2006), and few studies have reported no associations (Hirsch et al. 1999; Venn et al. 2000).

This study made it possible to include exposure from birth to asthma onset, measured at the historical residential addresses of each child. We used a dispersion model based on prospectively collected data for several time periods including time variations in emissions and detailed measurements of meteorologic data and background air pollution, presenting a longitudinal design (Oftedal et al. 2008). Another well-designed study is a French study using traffic exposure on updated addresses during each child’s life (Zmirou et al. 2004). This French study has reported associations with recent asthma diagnosis for early exposure, but not for lifetime exposure. A cohort study from Japan has reported no associations with asthma in first-graders, but with incident asthma using NO_2_ measurements from the nearest monitoring station to each school (Shima et al. 2002). Another recent Scandinavian cohort study has reported no associations with asthma diagnosis combined with current wheeze in preschool children using a similar exposure model as in our study (Nordling et al. 2008). A recent cohort study from California has reported associations with incident asthma diagnosis using outside home measurements of NO_2_ determined after asthma onset (Jerrett et al. 2008). In contrast to our study, most studies have used asthma diagnosis ever without time for onset. Of these studies, a study from California and two large Taiwanese studies have reported associations at higher exposure levels than our study (Gauderman et al. 2005; Guo et al. 1999; Hwang et al. 2005), and others at comparable levels (Brauer et al. 2007; Gordian et al. 2006). At similar exposure levels as our study, some studies have reported associations in subpopulations (Brauer et al. 2002; Hirsch et al. 1999). However, some well-designed studies among children exposed to higher levels have not reported positive associations (Diette et al. 2007; Krämer et al. 2000) or have reported associations in subpopulations only (Clougherty et al. 2007; McConnell et al. 2002, 2006; Nicolai et al. 2003). Several of these studies used exposure metrics determined after asthma onset (Gauderman et al. 2005; Gordian et al. 2006) or probably after asthma onset (Brauer et al. 2002, 2007; Diette et al. 2007; Guo et al. 1999; Hirsch et al. 1999; Hwang et al. 2005; Krämer et al. 2000; McConnell et al. 2002; Nicolai et al. 2003; Shima et al. 2002). Thus, it is not clear whether air pollution exposure can induce development of asthma, and we speculate that higher levels of exposure than was present in Oslo may be needed. However, in observational studies other issues also need to be considered.

Not all eligible children were willing to participate in the study, and the participation rate was higher in the cohort than in the noncohort population. These populations differed in several ways: The cohort population had more atopic, more western, and more educated parents and lived at addresses with lower NO_2_ levels than did the noncohort population. Thus, parents with nonasthmatic children may have participated to a lesser extent than parents with asthmatic children. The prevalence of parental atopy is comparable to that in the PIAMA (The Prevention and Incidence of Asthma and Mite Allergy) study (Brauer et al. 2002), although considerably higher than in the Swedish cohort (Nordling et al. 2008), and may have increased the reported rates of asthma and respiratory symptoms. Ethnicity was different in the two populations: is study population of schoolchildren may be slightly less nonwestern than the overall nonwestern population in Oslo (15%), which comprises more children than adults (Aalandslid and Østby 2007). Related to ethnicity is education, and the education levels are high and probably higher in the study population than among those invited. Further, it is reasonable to assume that more highly educated parents are more likely to visit their doctor when their child gets ill and may be more eager to get a diagnosis than parents with less education. Combined with lower NO_2_ levels in the highly educated cohort population, this factor may have produced more asthma diagnoses at lower NO_2_ levels than at higher levels. We cannot exclude that the consequence of this likely selection problem may have distorted the associations, and it may explain the negative association in the cohort population.

Assessing asthma by questionnaire has limitations, although self-reported doctor-diagnosed asthma has been reported to reflect what doctors actually told their patients, at least among adults, and validity assessed by repeatability of response has been reported to be good (Ehrlich et al. 1995). Thus, self-reported doctor diagnosis has been widely used in epidemiologic studies, and has been recommended as the preferred outcome for use in large population-based studies, because a more accurate diagnosis is not available (Burr 1992). Asthma diagnosis in early lifetime represents early symptoms which to some extent may indicate asthma, and is often related to respiratory tract infections (Villeneuve et al. 2007), in contrast to the atopy-related diagnosis in older children (Martinez et al. 1995). This is confirmed by the data on SPT positivity, with more sensitized children of those with later asthma onset than those with early onset. Most of the diagnoses were reported in early lifetime, and a considerable proportion of the children had recovered at 4 years of age. Including early onset cases who recovered during follow-up as noncases also produced less negative associations with NO_2_ exposure. Distance to major road was not associated with asthma onset, which confirms the findings of modeled exposure. Interestingly, in contrast to the negative association for early asthma onset, the association between NO_2_ exposure and late asthma onset showed a positive, though not statistically significant trend (due to very few cases), and the CIs overlapped. Lack of power may have contributed to the null findings of late onset, where diagnostic accuracy probably is higher. The negative associations do not seem plausible and may be explained by selective or different practice of diagnosing asthma in early life that for some reason seems to be related to where the children lived. In Oslo, people usually visit a doctor in their neighborhood, and doctors may have different diagnostic practices depending on their location. That children with early diagnosis more often had western and highly educated parents and probably lived in less-polluted areas compared to those with late diagnosis suggests that cultural differences, parental eagerness, and resources may have contributed. This possible misclassification may have produced more negative associations, although it is unlikely that the associations could have been reversed.

Age for asthma onset was retrospectively assessed, and parental recall fades with time. However, the findings of the logistic regression analyses that ignored time of onset confirmed the results of proportional hazard modeling. Thus, parental recall is not likely to have significantly affected the accuracy of time to asthma. This potential recall error should be independent of the exposures, and random error would probably dilute the association between exposure and outcome.

Moving out of Oslo because of asthma onset was not an issue in this study population, and measurement problems stemming from moving within the city are captured by exposure before asthma onset. Besides, residential outdoor exposure does not measure true individual exposure, and air pollution modeling may produce error in the exposure estimates (Oftedal et al. 2007). More specific, less reliable emissions and fewer receptor points with busy traffic in the early period lead to more uncertain modeling. However, because the spatial variation of air pollution is mostly controlled by the meteorologic and topographic conditions in Oslo (Gram et al. 2003; Oftedal et al. 2009), we believe that the ordering of residential NO_2_ exposures was maintained over time. Because all traffic-related exposures were calculated independently from health outcomes and potential confounders, systematic error is unlikely, and random error would probably dilute associations between exposure and outcome.

We cannot exclude the possibility of unmeasured confounders even though we have adjusted for several potential risk factors for asthma including socioeconomic factors, which did not influence the estimated associations. We also adjusted for several socioeconomic variables on the neighborhood level, none of which affected the associations.

The associations between previous year’s residential exposure and the current asthma-related symptoms tended to be positive, although not statistically significant. Precise information on timing of symptom occurrence was not recorded. Thus, it was not possible to assess short-term effects of traffic-related exposures in an optimal way, and may explain why we did not identify statistically significant associations.

NO_2_ and particulate matter are traffic-related pollutants and the modeled concentrations are highly correlated in Oslo. NO_2_ is not considered a causal agent for respiratory effects, but may in this study act as a general assessment of traffic-related air pollution. The other exposure, distance to major road, represents exposure to traffic but may also express socioeconomic status (Nicolai et al. 2003; Vineis et al. 2007), which entails difficulties in separating these effects (Nicolai et al. 2003).

In this population-based study in 9- to 10-year-old children in Oslo, we applied modeled NO_2_ exposure at historical home addresses from birth to asthma onset and distance to major road for each child. We were not able to find positive associations between any long-term traffic-related exposure and development of asthma. These results may be explained by a likely selection problem combined with diagnostic misclassification of early asthma, but we have little reason to believe that the associations could have been reversed. Alternatively, one may speculate that the exposure levels may have been too low to reveal notable positive associations.

## Figures and Tables

**Table 1:** Characteristics of the study population.

Characteristic	All subjects with questionnaire information (*n* = 3,533)	Subjects with both traffic-related exposures and questionnaire information		
		Total population (*n* = 2,871)	Cohort population (*n* = 1,551)	Noncohort population (*n* = 1,320)
Sex (percent male)	50.9	51.1	50.2	52.0
Age [years (mean ± SD)]	9.36 ± 0.37	9.35 ± 0.36	9.28 ± 0.35	9.45 ± 0.35
Birth weight [kg (mean ± SD)]^a^	3.51± 0.55	3.50 ± 0.56	3.57 ± 0.51	3.40 ± 0.61
Furry pets in early life (%)	18.1	18.1	19.3	16.7
Wall-to-wall carpeting in early life (%)	31.0	32.3	31.5	33.2
Dampness problems in early life (%)^a^	6.3	5.7	4.7	7.0
Parental ethnicity western only (%)^b^	75.9	79.2	92.2	63.4
Parental atopy (%)	57.1	59.3	64.0	53.7
Maternal smoking in pregnancy (%)	15.5	16.1	15.3	17.2
Maternal education (%)				
9–12 years	28.4	29.4	28.2	31.0
> 12 years	59.1	58.6	65.9	49.3
Paternal education (%)				
9–12 years	25.0	25.5	23.3	28.1
> 12 years	60.8	60.3	66.9	52.1
Maternal marital status (%)^a^				
Cohabitant	28.1	28.0	30.8	23.7
Single	8.8	7.6	5.5	10.5
Cohort indicator (%)	44.6	54.0	100	0
Skin prick test positivity (%)^a^	24.0	23.9	23.5	24.4
Furry pets now (%)	30.0	31.2	35.0	26.7
Wall-to-wall carpeting now (%)	13.0	13.2	10.9	16.0
Parental smoking now (%)	18.1	17.8	14.0	22.3

a Maximum number of subjects with missing data for boys/girls was 395/400 (SPT positivity) and 274/305 (maternal marital status as next covariate after SPT positivity) for the subjects with questionnaire information, 306/306 (SPT positivity), and 135/133 (birth weight as next covariate after SPT positivity) for the subjects with both traffic-related exposures and questionnaire information.

b Western ethnicity was defined as western Europe, United States, Canada, Australia, or New Zealand as birth country.

**Table 2:** Distribution of long-term traffic-related exposure levels for 9- to 10-year-old children (*n* = 2,871).

Exposure metric	Min	25th percentile	Median	Mean	75th percentile	Max
NO_2_ 1st year of life (μg/m^3^)	1.5	24.9	40.5	39.3	52.8	84.0
NO_2_ 2nd year of life (μg/m^3^)^a^	1.7	18.8	31.8	31.1	42.9	70.6
NO_2_ previous year (μg/m^3^)	1.4	15.7	25.3	25.2	33.7	65.1
Distance to major road at birth address (m)	4.5	217.1	403.6	576.4	740.7	9592.2
Distance to major road at 10 years (m)^b^	4.9	245.1	458.5	629.0	802.3	9592.2

Abbreviations: Max, maximum; Min, minimum.

a Eleven boys and 13 girls had missing NO_2_ levels in second year of life.

b Distance to major road at address for date of completing the questionnaire.

**Table 3:** Incidence, RR (95% CI), and OR (95% CI) of doctor-diagnosed asthma onset in 9- to 10-year-old children (*n* = 2,329), for an IQR increase of long-term traffic-related exposure.

Doctor-diagnosed asthma onset	Exposure metric	IR/person-year (no. of cases)	RR per IQR^a^ increase		OR per IQR^a^ increase adjusted^d^
			Unadjusted^b^	Adjusted^c^	
Any yearly onset	NO_2_ 1st year of life	0.014 (274)	0.85 (0.69–1.04)	0.82 (0.67–1.02)	0.81 (0.65–1.02)
Any yearly onset	NO_2_ before onset	0.014 (274)	0.85 (0.70–1.05)	0.82 (0.67–1.02)	—
Any yearly onset	Distance to major road^e^	0.014 (274)	0.99 (0.91–1.08)	0.99 (0.90–1.08)	0.98 (0.89–1.08)
Early onset (< 4 years of age)	NO_2_ before onset	0.034 (226)	0.81 (0.64–1.01)	0.78 (0.62–0.98)*	—
Early onset (< 4 years of age)	Distance to major road^e^	0.034 (226)	0.99 (0.90–1.09)	0.98 (0.89–1.08)	0.98 (0.88–1.09)
Late onset (≥ 4 years of age)^f^	NO_2_ before onset	0.004 (48)	1.12 (0.68–1.84)	1.05 (0.64–1.72)	—
Late onset (≥ 4 years of age)^f^	Distance to major road^e^	0.004 (48)	1.02 (0.80–1.30)	1.00 (0.79–1.26)	1.00 (0.79–1.27)

CI, confidence interval.

a IQR for NO_2_ varies between 27.3 and 19.6 μg/m^3^, decreasing over time. For distance to major road IQR is −540.6 m, the difference between 25th percentile and 75th percentile (i.e., living closer compared to living farther from the major road).

b Cox proportional hazard regression.

c Cox proportional hazard regression model adjusted for sex, parental atopy, paternal education, maternal smoking in pregnancy, maternal marital status at the child’s birth and indicator for cohort population.

d Logistic regression ignored timing of asthma onset and the same covariates as in the proportional hazard regression were adjusted for.

e Distance to major road at birth address.

f Children with early asthma onset and children censored before four years of age were excluded.

* *p* < 0.05.

**Table 4:** Prevalence (%) and OR (95% CI) of current respiratory symptoms in 9- to 10-year-old children (*n* = 2,205) for an IQR increase of long-term traffic-related exposure.

Respiratory symptoms	Exposure metric	Percent prevalence^a^ (no. of cases)	OR per IQR^b^ increase	
			Unadjusted^c^	Adjusted^d^
Current wheeze	NO_2_ previous year	14.5 (319)	1.06 (0.88–1.28)	1.01 (0.83–1.23)
Current wheeze	Distance to major road^e^	14.5 (319)	1.03 (0.93–1.14)	1.00 (0.91–1.11)
Current severe wheeze	NO_2_ previous year	4.8 (106)	1.11 (0.82–1.52)	1.10 (0.79–1.51)
Current severe wheeze	Distance to major road^e^	4.8 (106)	0.97 (0.84–1.13)	0.96 (0.82–1.11)
Current dry cough	NO_2_ previous year	19.3 (410)	1.08 (0.90–1.28)	1.01 (0.84–1.21)
Current dry cough	Distance to major road^e^	19.3 (410)	1.04 (0.95–1.15)	1.02 (0.93–1.12)

a *n* = 2,201 for current severe wheeze and *n* = 2,129 for current dry cough.

b IQR for NO_2_ is 17.9 μg/m^3^ for current wheeze and for current severe wheeze (*n* = 2,201), and 18.1 μg/m^3^ for current dry cough (*n* = 2,129). For distance to major road IQR is the difference between 25th and 75th percentile (i.e., living closer vs. living farther from the major road). IQR is −580.3 m for current wheeze, −580.9 m for current severe wheeze (*n* = 2,201), and −576.6 m for current dry cough (*n* = 2,129).

c Simple logistic regression.

d Logistic regression model adjusted for sex, parental ethnicity and atopy, paternal education, maternal smoking in pregnancy, maternal marital status at the child’s birth, indicator for cohort population, keeping furry pets now, and dampness problems now.

e Distance to major road at address for date of completing the questionnaire.

## References

[b1-ehp-117-839] Aalandslid V, Østby L (2007). Innvandrermangfold i Kommune-Norge. Få har mange, mange har få [in Norwegian]. Samfunnsspeilet nr.4.

[b2-ehp-117-839] Brauer M, Hoek G, Smit HA, de Jongste JC, Gerritsen J, Postma DS (2007). Air pollution and development of asthma, allergy and infections in a birth cohort. Eur Respir J.

[b3-ehp-117-839] Brauer M, Hoek G, van Vliet P, Meliefste K, Fischer PH, Wijga A (2002). Air pollution from traffic and the development of respiratory infections and asthmatic and allergic symptoms in children. Am J Respir Crit Care Med.

[b4-ehp-117-839] Burr ML (1992). Diagnosing asthma by questionnaire in epidemiological surveys. Clin Exp Allergy.

[b5-ehp-117-839] Clougherty JE, Levy JI, Kubzansky LD, Ryan PB, Suglia SF, Canner MJ (2007). Synergistic effects of traffic-related air pollution and exposure to violence on urban asthma etiology. Environ Health Perspect.

[b6-ehp-117-839] Delfino RJ (2006). Think globally, breathe locally. Thorax.

[b7-ehp-117-839] Diette GB, Hansel NN, Buckley TJ, Curtin-Brosnan J, Eggleston PA, Matsui EC (2007). Home indoor pollutant exposures among inner-city children with and without asthma. Environ Health Perspect.

[b8-ehp-117-839] Eder W, Ege MJ, von Mutius E (2006). The asthma epidemic. N Engl J Med.

[b9-ehp-117-839] Ehrlich RI, Toit DD, Jordaan E, Volmink JA, Weinberg EG, Zwarenstein M (1995). Prevalence and reliability of asthma symptoms in primary school children in Cape Town. Int J Epidemiol.

[b10-ehp-117-839] Gauderman WJ, Avol E, Lurmann F, Kuenzli N, Gilliland F, Peters J (2005). Childhood asthma and exposure to traffic and nitrogen dioxide. Epidemiology.

[b11-ehp-117-839] Gordian ME, Haneuse S, Wakefield J (2006). An investigation of the association between traffic exposure and the diagnosis of asthma in children. J Expo Sci Environ Epidemiol.

[b12-ehp-117-839] Gram F, Nafstad P, Håheim LL (2003). Estimating residential air pollution exposure among citizens in Oslo 1974–1998 using a geographical information system. J Environ Monit.

[b13-ehp-117-839] Guo YL, Lin YC, Sung FC, Huang SL, Ko YC, Lai JS (1999). Climate, traffic-related air pollutants, and asthma prevalence in middle-school children in Taiwan. Environ Health Perspect.

[b14-ehp-117-839] Heinrich J, Wichmann HE (2004). Traffic related pollutants in Europe and their effect on allergic disease. Curr Opin Allergy Clin Immunol.

[b15-ehp-117-839] Hirsch T, Weiland SK, von Mutius E, Safeca AF, Gräfe H, Csaplovics E (1999). Inner city air pollution and respiratory health and atopy in children. Eur Respir J.

[b16-ehp-117-839] Hwang BF, Lee YL, Lin YC, Jaakkola JJK, Guo YL (2005). Traffic related air pollution as a determinant of asthma among Taiwanese school children. Thorax.

[b17-ehp-117-839] Jerrett M (2007). Does traffic-related air pollution contribute to respiratory disease formation in children?. Eur Respir J.

[b18-ehp-117-839] Jerrett M, Shankardass K, Berhane K, Gauderman WJ, Künzli N, Avol E (2008). Traffic-related air pollution and asthma onset in children: a prospective cohort study with individual exposure measurement. Environ Health Perspect.

[b19-ehp-117-839] Krämer U, Koch T, Ranft U, Ring J, Behrendt H (2000). Traffic-related air pollution is associated with atopy in children living in urban areas. Epidemiology.

[b20-ehp-117-839] Kuehni CE, Strippoli MP, Zwahlen M, Silverman M (2006). Association between reported exposure to road traffic and respiratory symptoms in children: evidence of bias. Int J Epidemiol.

[b21-ehp-117-839] Lewis SA, Antoniak M, Venn AJ, Davies L, Goodwin A, Salfield N (2005). Secondhand smoke, dietary fruit intake, road traffic exposures, and the prevalence of asthma: a cross-sectional study in young children. Am J Epidemiol.

[b22-ehp-117-839] Martinez FD, Wright AL, Taussig LM, Holberg CJ, Halonen M, Morgan WJ (1995). Asthma and wheezing in the first six years of life. N Engl J Med.

[b23-ehp-117-839] McConnell R, Berhane K, Gilliland F, London SJ, Islam T, Gauderman WJ (2002). Asthma in exercising children exposed to ozone: a cohort study. Lancet.

[b24-ehp-117-839] McConnell R, Berhane K, Yao L, Jerrett M, Lurmann F, Gilliland F (2006). Traffic, susceptibility, and childhood asthma. Environ Health Perspect.

[b25-ehp-117-839] Næss O, Piro FN, Nafstad P, Smith GD, Leyland AH (2007). Air pollution, social deprivation, and mortality: a multilevel cohort study. Epidemiology.

[b26-ehp-117-839] Nafstad P, Brunekreef B, Skrondal A, Nystad W (2005). Early respiratory infections, asthma, and allergy: 10-year follow-up of the Oslo Birth Cohort. Pediatrics.

[b27-ehp-117-839] Nicolai T, Carr D, Weiland SK, Duhme H, von Ehrenstein O, Wagner C (2003). Urban traffic and pollutant exposure related to respiratory outcomes and atopy in a large sample of children. Eur Respir J.

[b28-ehp-117-839] Nordling E, Berglind N, Melén E, Emenius G, Hallberg J, Nyberg F (2008). Traffic-related air pollution and childhood respiratory symptoms, function and allergies. Epidemiology.

[b29-ehp-117-839] Oftedal B, Brunekreef B, Nystad W, Nafstad P (2007). Residential outdoor air pollution and allergen sensitization in schoolchildren in Oslo, Norway. Clin Exp Allergy.

[b30-ehp-117-839] Oftedal B, Walker SE, Gram F, McInnes H, Nafstad P (2009). Modelling long-term averages of local ambient air pollution in Oslo, Norway: evaluation of nitrogen dioxide, PM_10_ and PM_2.5_. Int J Environ Pollut.

[b31-ehp-117-839] Pierse N, Rushton L, Harris RS, Kuehni CE, Silverman M, Grigg J (2006). Locally generated particulate pollution and respiratory symptoms in young children. Thorax.

[b32-ehp-117-839] Sarnat JA, Holguin F (2007). Asthma and air quality. Curr Opin Pulm Med.

[b33-ehp-117-839] Shima M, Nitta Y, Ando M, Adachi M (2002). Effects of air pollution on the prevalence and incidence of asthma in children. Arch Environ Health.

[b34-ehp-117-839] U.S. EPA (2004). Air Quality Criteria for Particulate Matter.

[b35-ehp-117-839] Vassenden K (1987). Folke- og boligtellingene 1960, 1970 og 1980 [in Norwegian]. Rapporter fra Statistisk sentralbyrå 87/2.

[b36-ehp-117-839] Venn A, Lewis S, Cooper M, Hubbard R, Hill I, Boddy R (2000). Local road traffic activity and the prevalence, severity, and persistence of wheeze in school children: combined cross sectional and longitudinal study. Occup Environ Med.

[b37-ehp-117-839] Villeneuve PJ, Chen L, Rowe BH, Coates F (2007). Outdoor air pollution and emergency department visits for asthma among children and adults: a case-crossover study in northern Alberta, Canada. Environ Health.

[b38-ehp-117-839] Vineis P, Hoek G, Krzyzanowski M, Vigna-Taglianti F, Veglia F, Airoldi L (2007). Lung cancers attributable to environmental tobacco smoke and air pollution in non-smokers in different European countries: a prospective study. Environ Health.

[b39-ehp-117-839] Zmirou D, Gauvin S, Pin I, Momas I, Sahraoui F, Just J (2004). Traffic related air pollution and incidence of childhood asthma: results of the Vesta case-control study. J Epidemiol Community Health.

